# A Chromosome-Level Genome Assembly and Resequencing Data Reveal Low DNA Methylation and Reduced Diversity in the Solitary Bee Pollinator *Osmia cornuta*

**DOI:** 10.1093/gbe/evaf224

**Published:** 2026-01-08

**Authors:** Jannik S Möllmann, Xuejing Hu, Eva A Baumgarten, Anne Hartleib, Katja Nowick, Thomas J Colgan, Eckart Stolle

**Affiliations:** Institute of Organismic and Molecular Evolution, Johannes Gutenberg University, Mainz 55128, Germany; Leibniz Institute for the Analysis of Biodiversity Change, Museum Koenig, Centre for Molecular Biodiversity Research, Bonn 53113, Germany; Institute of Organismic and Molecular Evolution, Johannes Gutenberg University, Mainz 55128, Germany; Institute of Biology, Freie Universität Berlin, Berlin 14195, Germany; Institute of Biology, Freie Universität Berlin, Berlin 14195, Germany; Institute of Organismic and Molecular Evolution, Johannes Gutenberg University, Mainz 55128, Germany; Institute of Organismic and Molecular Evolution, Johannes Gutenberg University, Mainz 55128, Germany

**Keywords:** *Osmia cornuta*, solitary bees, genome assembly, gene prediction, DNA methylation, genetic diversity

## Abstract

Bees provide essential pollination services that contribute to ecosystem stability, as well as the sustainability of economic crop yields. Due to concerns over global and local declines, improving our understanding of these ecologically and commercially important species is crucial for determining their capacity to respond and adapt to environmental challenges. The European orchard bee (*Osmia cornuta*) is a solitary bee of increasing agricultural importance due to its role in the pollination of fruit crops, yet lacks genomic resources. Using cost-effective Nanopore-only long-read sequencing, we report the first genome assembly for *O. cornuta*, spanning 647.56 Mb across 727 contigs (N50 = 3.94 Mb) at a high level of completeness (99.88% BUSCO complete). In line with the expected number of chromosomes in this species, 16 major scaffolds were assembled to chromosome level. Also, we provisionally investigated the epigenomic architecture of *O. cornuta*, finding low numbers of CG dinucleotides that were either 5′-methylated or 5′-hydroxymethylated, providing additional evidence for the limited role methylation plays in gene regulation in Hymenopterans. To generate improved gene annotations, we combined transcriptomic- and orthology-based approaches, leading to the prediction of 12,144 genes and 25,964 proteins, showing exceptionally high BUSCO completeness (99.64%). Lastly, through whole-genome resequencing of a representative dataset, we provisionally find patterns of reduced nucleotide diversity and lower recombination rates within *O. cornuta* compared to other bee species. Collectively, our study provides a novel insight into the genome architecture of a key pollinator, providing an important resource to facilitate further genomic studies.

Significance Solitary bees are crucial pollinators for crops and wild plants, but scientists have lacked the genetic blueprints needed to understand how these species might adapt to environmental threats like climate change and habitat loss. Using modern and cost-effective technologies, we generated the complete genome of the European orchard bee, an economically important fruit pollinator. This comprehensive genetic resource provides the foundation for future research on solitary bee biology and conservation, necessary to understand their adaptive potential in the face of environmental challenges. In line with this, we analyzed this species’ genetic variation across populations in Germany using a small, representative dataset and found surprisingly low genetic diversity and limited evidence of gene recombination during reproduction, which are factors that could restrict its capacity to evolve in response to environmental challenges.

## Introduction

Improving our fundamental understanding of the biology of ecologically and commercially important pollinators is particularly important considering they provide key ecosystem services that are crucial for ecosystem stability, biodiversity, and sustainability of agricultural yields (Kremen et al. 2007; Gallai et al. 2009; Potts et al. 2016). Despite this, declines in natural populations have raised concerns about the long-term viability of such pollination services (Hallmann et al. 2017; Powney et al. 2019; Lippert et al. 2021). Key factors that are proposed to contribute to declines include habitat loss and fragmentation, pesticides, parasites and pathogens, invasive species, and changes in climatic conditions (Cameron et al. 2011; Potts et al. 2016; Seibold et al. 2019; Möllmann et al. 2024). In addition, for certain pollinators, such as solitary bees, the lack of social buffering commonly found in social bee colonies may mean they are predicted to be even more susceptible to environmental challenges that contribute to declines (Straub et al. 2015). It is, thus, of vital importance to improve our understanding of the biology of solitary bees to inform on their capacity to respond and adapt to environmental challenges.

An important solitary bee of ecological and commercial importance is the European orchard bee *Osmia cornuta* (Latreille, 1805), which is native to most of Central and Southern Europe with its range extending south to Northern Africa and east to Central Asia (Banaszak and Romasenko 1998). It belongs to a taxon-rich genus of over 300 species (Praz et al. 2008), which are part of the family Megachilidae. Due to their high pollination efficiency in fruit orchards, mason bees, such as *O. cornuta*, have seen increasing commercial use, which has also led to an increased presence in scientific studies (Gruber et al. 2011; Sedivy and Dorn 2014; Fliszkiewicz and Giejdasz 2023). However, they also contain certain traits, such as a univoltine life cycle, generalist foraging patterns, and generalist nesting behavior (Splitt et al. 2022), which make them an increasingly suitable model to study and understand aspects that are more common to the biology of solitary bees (Bosch 1994; Fauria and Campan 1998; Vicens and Bosch 2000; Bosch and Kemp 2004; Bosch and Vicens 2006; Strobl et al. 2019). *Osmia cornuta* has also raised recent ecological concerns over its impact through accidental introductions into locations where it is non-native. For example, cocoons of *O. cornuta* have been traded to North America and other places outside of Europe where recent reports have confirmed the presence of feral populations that have begun to establish, such as in British Columbia, Canada (Getz et al. 2024).

Recent advances in sequencing technologies and the accompanying development of genomic tools to analyze these data have facilitated the rapid generation of genetic data that has informed on the genomic architecture of non-model organisms, including solitary bees (Barribeau et al. 2015; Kapheim et al. 2015, 2019; Beadle et al. 2019; Möllmann and Colgan 2022; Stolle et al. 2023; Santos et al. 2025; Wang et al. 2025; Yang et al. 2025), which has the potential for application in the fields of agriculture, as well as conservation and wildlife management approaches (Allendorf et al. 2010; Trapp et al. 2017; Kelemen and Rehan 2021). For example, through the generation of whole-genome sequencing data, it is now possible to accurately determine millions of nucleotide bases providing a solid basis to allow for estimating genetic parameters relevant for ecological modeling and conservation biology, such as genetic variation, effective population size and population connectivity, or identify regions of the genome with signatures of selection, helping to understand the adaptive potential of these species (Eizaguirre and Baltazar-Soares 2014; Webster et al. 2023). Similarly, transcriptomic-based methods enable the inference of accurate gene models as the basis for the identification of candidate genes and molecular pathways involved in responses to environmental stressors, such as climate change and pesticide exposure (Beadle et al. 2019; Möllmann and Colgan 2022).

In order to leverage these approaches, however, the availability of a high-quality chromosome-level genome assembly, including accurately predicted gene models, is of utmost importance as it underpins all genetic and transcriptomic analyses performed. For a few members of the genus *Osmia*, genome assemblies are already available (Beadle et al. 2019); however, some of these were assembled using short-read data, meaning they are likely less contiguous and more fragmentary, and may suffer from lower quality gene model predictions, limiting their utility compared to long-read generated chromosome assemblies (Ouyang et al. 2025; Wang et al. 2025). Therefore, for *Osmia* species, such as *O. cornuta*, improvements in the quality of genome assembly, which can be generated to chromosome level using long-read sequencing data, are urgently warranted to improve our understanding of the genetic architecture of these species, as well as provide sufficient resources to profile the genetic variation harbored in natural populations and resolve aspects of their recent demographic history. Here, we present a genome-based analysis of *O. cornuta*, for which we sequenced an individual male to high sequencing depth using Oxford Nanopore long-read sequencing technology, which we subsequently assembled to chromosome-level and generated high-quality gene model predictions using a combination of transcriptomic- and orthology-based gene annotation approaches. We also provide insights into the epigenomic landscape of *O. cornuta* through examination of DNA methylation levels, genome complexity through analysis of repeat diversity, as well as perform provisional analyses of nucleotide diversity and recombination rates through whole-genome sequencing of individuals sampled from a European population.

## Results and Discussion

### High-completeness, Chromosome-Level Genome Assembly From Long-Read Sequencing Data

**Fig. 1.**
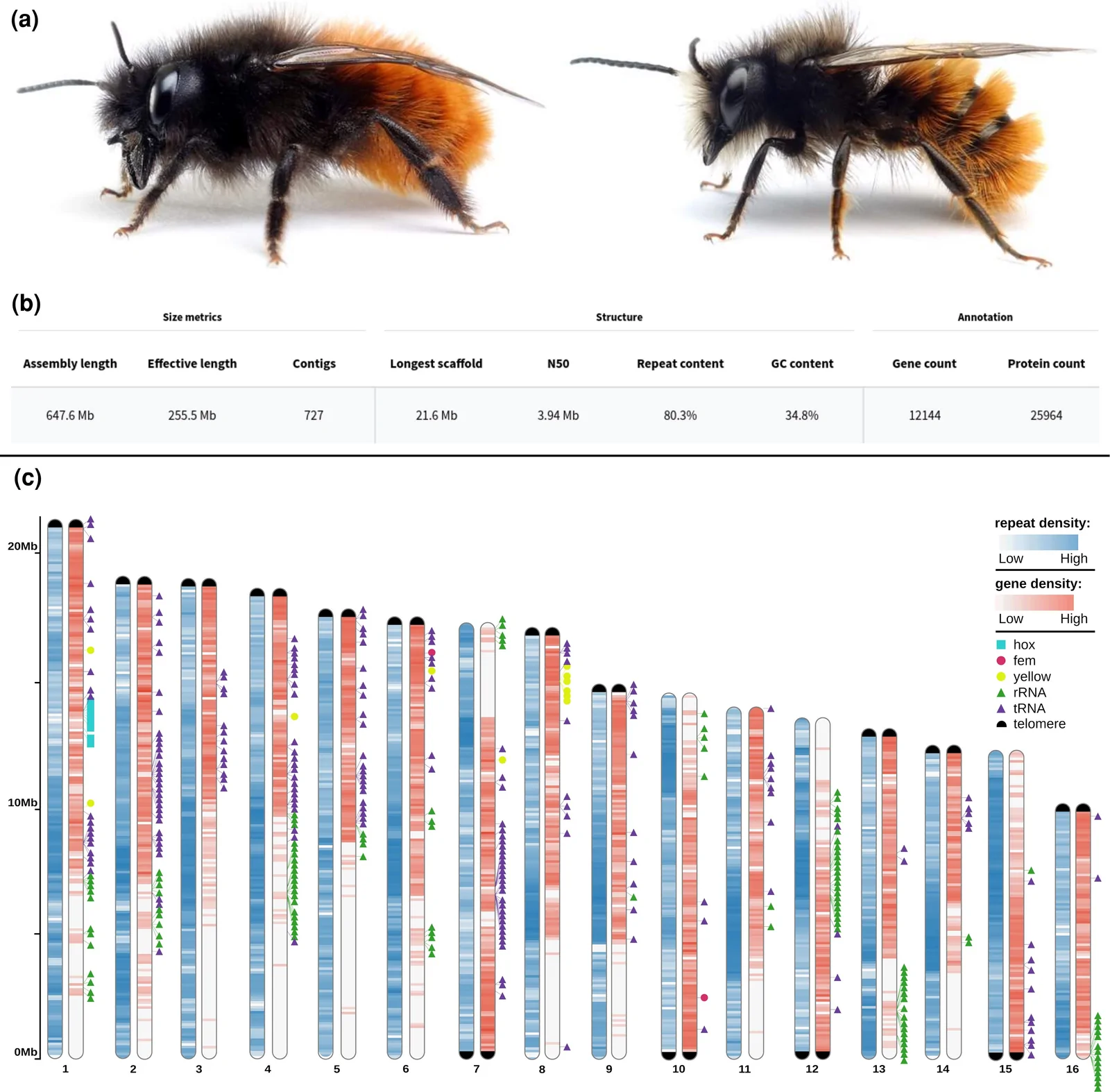
Habitus, overview statistics and schematic chromosomal karyogram. a) Habitus of *Osmia cornuta*, depicting both a female (left) as well as a male (right) individual (image copyright: Mandy Firtzsche www.apidarium.de). b) Assembly and annotation summary statistics, classified into size-related, assembly structure-related, and annotation-related metrics. c) A schematic karyogram that depicts for each of the 16 chromosomes regions of increased or reduced gene- and repeat-density using color spectra. Depicted pairs of chromosomes do not represent separate, diploid chromosome homologs but copies of the same haploid chromosome, each copy highlighting the density of a different set of genetic features. In addition, the presence and location of some representative gene families, which tend to cluster in specific regions of the genome, are depicted using colored symbols. An additional, black symbol is used to mark telomeres at chromosomal ends. The size of this symbol is uniform and does not represent the actual length of these telomeric regions.

**Fig. 2.**
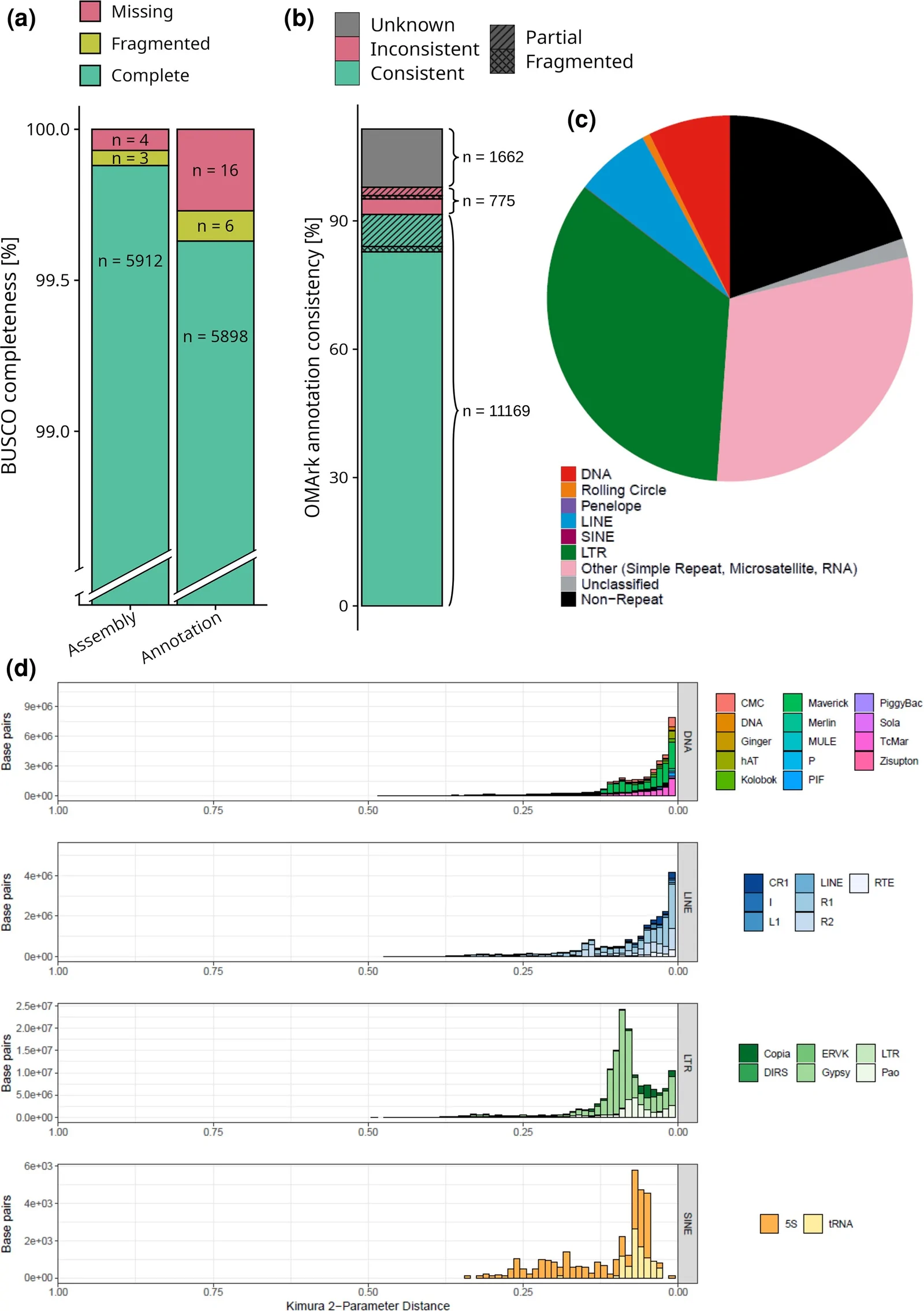
High assembly- and annotation completeness and overview of genomic repeat architecture. a) Stacked bar charts showing the results of the overlap of the assembly (left) and the predicted protein models of the annotation (right) with single-copy orthologs from the hymenopteran dataset (*n* = 5,919) of the OrthoDB (v12) database. Within each bar chart, the level of overlap for three subcategories is color-coded: “Complete,” green, refers to an assembly region or a protein fully overlapping with an ortholog in the reference database; “Fragmented,” yellow, refers to an assembly region or a protein that partially overlaps with a reference ortholog; and “Missing,” red, which refers to a reference ortholog that did not overlap with any region of the assembly or predicted protein set. b) A bar chart showing the annotation consistency of the predicted gene models when compared against hierarchical orthogroups (HOGs) of the OMA database with subcategories either color-coded or patterned: “Consistent,” green, referring to a gene model has been placed in the correct lineage; “Inconsistent,” red, referring to a gene model that has been placed in an incorrect, unspecific lineage; “Unknown,” gray, which refers to a gene model has not been placed in any of the tested lineages; “Partial,” stripes, meaning not all parts of a gene model match a HOG; and “Fragmented,” checkered, meaning a gene model does not match all parts of a HOG. c**)** A pie chart showing the genomic composition by repeat type and the fraction of non-repeat regions. Collectively, more than three quarters of the genome are made up of repeat sequences with long terminal repeats (LTR) being the most common. Legend: DNA = DNA transposon; Rolling Circle = rolling circle transposon; Penelope = Penelope transposon; SINE = short interspersed nuclear element; LTR = long terminal repeat; and RNA = retrotransposon. d**)** Four histograms, each depicting the distribution of the Kimura 2-parameter distance in base pairs for a specific type of repeat. Within each histogram, the repeat type is broken up into subtypes, each displayed at a different color.

From a single *O. cornuta* male (Fig. 1a), we generated 1,338,458 highly accurate, filtered reads on approximately half of a Nanopore PromethION flow cell at a total length of 26.12 Gb (mean length = 19.52 kb; maximum length = 930.51 kb). Filtered reads were then corrected using HERRO, resulting in 1,110,145 reads at a total length of 20.94 Gb (mean length = 18.86 kb; maximum read length = 139.28 kb). Using these reads, we generated 11 candidate assemblies, six of which were generated using flye and five were generated using hifiasm. Based on variation in contiguity, completeness, and the presence and extent of telomeres, we selected an assembly generated using hifiasm for further research and publication. This assembly initially contained 777 contigs (total length = 654.189 Mb), but was reduced by 44 contigs following purging of putative duplicate haplotigs. It had an excellent merqury QV score of 54.86, corresponding to a very low error rate (estimated error rate = 1 error per 306,000 bases), indicative of a high-quality, highly accurate assembly. Similarly, screens of foreign DNA did not classify any contigs as being contaminants. A scan for mitochondrial sequences suggested that six contigs were putative mitochondrial in origin. After removal of mitochondrial-derived contigs, the final assembly contained 727 contigs, without gaps, and a total length of 647,555,333 bp (Fig. 1b) with a BUSCO completeness score of 99.78% (duplicated: 0.12%, fragmented: 0.03%, missing: 0.07%) and a GC content of 34.8%. The longest contig spanned 21.64 Mb, the mean length of contigs was 890.72 kb, and N50, N70, and N90 lengths were 3.94 Mb (*n* = 29 contigs), 1.35 Mb (*n* = 91), and 0.34 Mb (*n* = 271), respectively. By mapping the reads back to the genome assembly, we inferred a mean coverage of 40.22× (SD = 32.14), whereas less repetitive parts of the largest primary contigs (*n* = 16) showed a coverage of approximately 55.14×. The high completeness of our assembly was further supported by the identification of 15 complete telomeres (AACCT) among the 16 largest contigs, which represent the 16 putative chromosomes previously described via karyotyping (Kerr and Laidlaw 1956). Another nine putative telomeres were found on shorter (<1.5 Mb) contigs. Additionally, we assembled the mitochondrion contig to a length of 35,525 bp, containing the expected two ribosomal RNA (rRNA), 24 transfer RNA (tRNA), and 13 protein-coding genes. A comprehensive annotation of repetitive elements determined that 80.3% of the genome was composed of repeats (Fig. 2c), of which the majority were LTR retrotransposons (34.29% of the assembly) and simple repeats (29.78%), while other types were much less common (DNA transposons: 7.25%, LINE: 6.67%, Rolling Circle: 0.6%, and Unclassified Repeats 1.7%). At 648 Mb, the length of this assembly is on the higher end, yet still very comparable to the length of publicly available high-quality genome assemblies in the same Genus (*Osmia bicornis*: 223 Mb, *Osmia lignaria*: 498.5 Mb) and Family (*Megachilda rotundata:* 613.4 Mb, *Coelioxys conoideus*: 417.6 Mb). Analogously, the BUSCO completeness score of 99.78% is similar, and even slightly higher, compared to high-quality assemblies of closely related species (*O. bicornis*: 98.5%, *O. lignaria*: 97.9%, see Table S2). Together, the identification of the presence and extent of telomeric regions and the exceedingly high BUSCO completeness suggest a highly contiguous genome assembly, comparable with high-quality PacBio and Hi-C genome assemblies of other bee species (Wallberg et al. 2019; Stolle et al. 2023; Toth et al. 2024; Wang et al. 2025).

### High-Quality Structural Annotations Generated Using a Combination of Protein-, Transcript-, and Orthology-Based Evidence

**Fig. 3.**
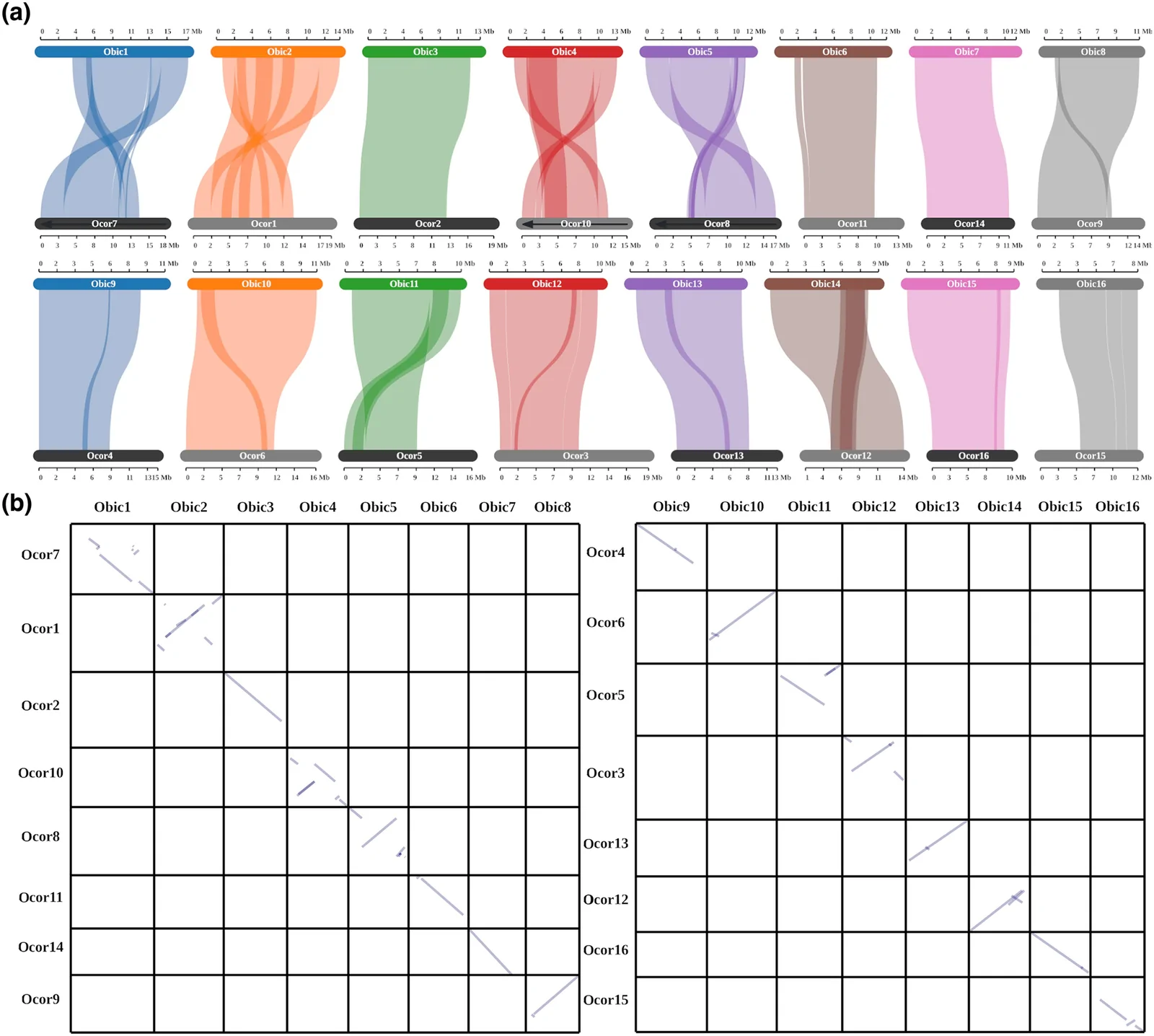
Synteny comparison with *Osmia bicornis*. a) A two-row parallel link plot giving an overview of syntenic regions between putative chromosomal scaffolds of *O. bicornis* (Obic; top of each row) and *O. cornuta* (Ocor: bottom of each row) based upon comparisons of predicted proteomes. The markers show the genomic coordinates for each putative chromosome in megabases (Mb). b) Dot plots displaying a more detailed examination of synteny between the two species. The binned *x* axis represents the position along identified chromosomes in *O. bicornis* with one chromosome per bin. Similarly, the *y* axis represents the position along chromosomes in *O. cornuta* with one chromosome per bin. A dot represents a syntenic region that is shared between the two species at the respective chromosomal loci.

We generated gene model predictions for the assembly using two independent approaches: first, using transcript- and protein-based evidence (implemented with BRAKER3); and second, using inferred orthology with the closely related *O. bicornis* (implemented with TOGA). We then combined predictions from these two approaches to generate a more complete set of gene models. The combined set of predictions comprised 12,144 genes, which is comparable to gene counts reported for long-read-based assemblies of related species (*O. bicornis:* 12,706; *O. lignaria:* 13,795; Table S2). The inferred gene set is predicted to encode for 25,946 protein-coding transcripts. The resulting protein-coding transcript predictions showed a BUSCO completeness score of 99.64%, whereas proteins from the individual annotation approaches resulted in completeness scores of 97.20% and 97.51%, respectively. Compared to the completeness score of 99.88% for the assembly (Fig. 2a), our high completeness score for the annotation suggests that, using this combined approach, we were able to predict gene models for nearly all genes that were assembled. The high completeness of the structural annotations was further highlighted by a high percentage (97.64%) of matches against conserved and taxonomically “consistent” hierarchical orthogroups (HOGs) from the Orthologous Matrix (OMA) database, as well as a low percentage (3.63%) of matches against HOGs that are considered taxonomically “inconsistent” with the species of interest (Fig. 2b). These numbers are highly comparable to the structural annotation of the closely related *O. bicornis* (<25 million years of divergence (Gonzalez 2011; Cardinal and Danforth 2013), 97.68% completeness, 3.67% “inconsistency” of proteome), which has been annotated via the NCBI Eukaryotic Genome annotation pipeline, indicating that our structural annotation has achieved a similar level of quality. In line with the high completeness of our gene annotations, we successfully confirmed the presence of several marked gene clusters and genes (Fig. 1c), among which the *yellow* (chromosome 8), *hox* (chromosome 1), and *osiris* clusters (chromosome 4), as well as the *feminizer* gene (chromosome 6), which forms part of the sex determining cascade. As our annotation approach did not predict noncoding gene models, we additionally scanned the chromosomal scaffolds of our assembly for tRNA and rRNA genes. In doing so, we found 195 tRNA genes with particularly pronounced clusters on chromosomes 2, 4, and 7, as well as 126 rRNA genes with strong clustering on chromosomes 4, 12, 13, and 16. Lastly, we also examined synteny of our genome assembly with that of the closely related *O. bicornis* via comparison of predicted proteomes. Summarized to gene level, we found that 77.43% of genes were collinear between the two species. Using this synteny-based approach, we further identified 16 chromosomal homologs to those described in *O. bicornis*, providing evidence of the high quality and completeness of the *O. cornuta* genome assembly. Additionally, we also found evidence of inverted segments on several chromosomes within the *O. cornuta* genome assembly, reflecting putative chromosomal rearrangements since the latest common ancestor (Fig. 3). In summary, our combined annotation approach produced a highly complete set of gene predictions for *O. cornuta*, successfully identifying key gene clusters, and demonstrating strong syntenic conservation with the closely related *O. bicornis*.

### Evidence of Genome-wide Methylation Patterns

A key advantage of Nanopore technology is its ability to detect modified bases, such as methylated cytosine, during sequencing, allowing for a direct examination of the epigenomic landscape. Methylation levels have previously been described as low in members of the Hymenoptera, including other bee species (Glastad et al. 2014; Bewick et al. 2017; Provataris et al. 2018), with studies employing bisulphite sequencing identifying approximately 1% of genome-wide cytosines reported as methylated in the honeybee *Apis mellifera* (Harris et al. 2019), and the bumblebee *Bombus terrestris* (Pozo et al. 2021). We, therefore, examined whether the genome of *O. cornuta* contained similar levels of methylated cytosines. By extracting base modification information directly from Nanopore data through basecalling, we found that the *O. cornuta* genome assembly contained no 6′-methyladenosine (6mA), 5′-methylcytosine (5mC), or 5′-hydroxymethylcytosine (5hmC) modifications, but low numbers of 5′-methylcytosine and 5′-hydroxymethylcytosine in the context of CG dinucleotides (5mCG, 5hmCG). For 5mCG, out of 24,194,970 called sites, 192,079 (0.84%) were considered methylated, being supported by at least one read, while 51,437 (0.23%) sites showed methylation within more than 20% of reads. For 5hmCG, out of 24,194,970 called sites, we identified 201,054 (0.9%) sites that were considered methylated, being supported by at least one read, while 14,125 sites (0.06%) showed methylation in more than 20% of reads. To understand how 5′-methylation of CpG loci varied across different regions and features of the genome, we inferred for each investigated genomic feature type, the percentage of sites that were considered weakly methylated, finding evidence of considerable variation in methylation patterns across the genome (Table 1). In particular, exons showed by far the highest proportion of 5′-methylated CpG sites (2.53%), while introns and intergenic regions had a relatively lower proportion of 5′-methylated CpG sites (intron: 0.88%, intergenic: 1.02%). Strikingly, in low complexity and repeat regions, the frequency of 5′-methylated CpG sites was up to 100× reduced compared to all other genomic feature types (low complexity: 0.01%; repeats: 0.03%). The same overall patterns were observed for 5′-hydroxymethylcytosine CpG modifications. Collectively, we found that DNA methylation levels within the genome of *O. cornuta* are low, however comparable to levels previously reported for other hymenopterans (Standage et al. 2016; Glastad et al. 2017; Oldroyd and Yagound 2021). As epigenetic modifications, such as DNA methylation, are linked to the adaptive potential of species (Ashe et al. 2021; Stajic and Jansen 2021), for example through their effect on phenotypic plasticity (Oostra et al. 2018; Fox et al. 2019; Romero-Mujalli et al. 2024), our findings of low methylation levels in this pollinator species raise concerns about its potential to adapt to future environmental challenges, such as climate change. In fact, this species’ previously reported increased susceptibility to pesticides after exposure to increased temperatures (Albacete et al. 2023) might be related to the herein reported low levels of methylation via their effect on phenotypic plasticity. However, the investigation of this hypothesis requires additional data and it is noteworthy that this species is currently undergoing population and range expansions (Westrich 2019), and thus does not seem to be limited by the herein reported epigenetic limitations, which raises interest in the genetic potential that allows this species to thrive.

**Table 1:** Genome-wide patterns of methylation across different genomic features

Genomic feature	5mCG			5hmCG		
	Methylated sites (%)	Methylated sites (*n*)	Methylatable sites (*n*)	Methylated sites (%)	Methylated sites (*n*)	Methylatable sites (*n*)
Exon	2.53	23,738	936,051	1.79	16,781	936,051
Intron	0.88	37,240	4,190,504	1.11	46,615	4,190,504
Intergenic	1.02	83,839	8,198,788	1.17	95,525	8,198,788
Low complexity	0.01	8654	7,448,992	0.02	12,157	7,448,992
Repeat	0.03	54,052	15,547,674	0.06	89,611	15,547,674

A table showing for each genomic feature considered in the analysis, the percentage as well as the number of CpG sites for which we found evidence of either a 5′-methylcytosine modification (5mCG) or 5′-hydroxymethylcytosine (5hmCG) modification. In addition, the total number of potentially methylated CpG sites for each genomic feature is shown in a separate column.

### Evidence of Reduced Genetic Diversity, Low Recombination Rates, and Reduced Effective Population Size Around the Last Glacial Maximum

As a preliminary examination of the population genetics of *O. cornuta* and as it has been reported that many groups within the Hymenoptera have relatively low genetic diversity and recombination rates compared to other insect groups (Weyna and Romiguier 2021), for example due to haplodiploidy (Zayed 2004; Zayed and Packer 2005; Suni and Hernandez 2023), we investigated genetic diversity, linkage disequilibrium, and recent changes in effective population size from whole-genome resequencing data for 10 females sampled from across Germany (sampling sites in Table S1). We identified 594,857 high-quality, filtered SNPs (biallelic: 99.7%) across the 16 chromosomal scaffolds of our assembly (255.5 Mb effective length), yielding an overall variant density of 2.33 SNPs per kilobase. Variant density varied across chromosomal scaffolds, ranging from 1.73 (Chromosome 3) to 3.22 SNPs per kilobase (Chromosome 15). Structural annotation revealed that the majority of variants (94.9%) were positioned in noncoding regions, such as intronic, intergenic, and regions upstream/downstream of genes. Among protein-coding regions, 74.5% of variants (*n* = 22,116) were synonymous (silent) polymorphisms preserving amino acid sequences, while 25.3% (*n* = 7,517) were nonsynonymous (missense) variants, predicted to alter the amino acid sequence of the resulting protein. Protein-truncating variants, such as early stop codons, were rare, with nonsense mutations attributed to only 0.14% (*n* = 42) of variants within protein-coding regions and critical splice site disruptions caused by only 21 variants genome-wide. The overall missense-to-silent ratio of 0.34 likely reflects the expected purifying selection acting against amino acid-altering mutations.

**Fig. 4.**
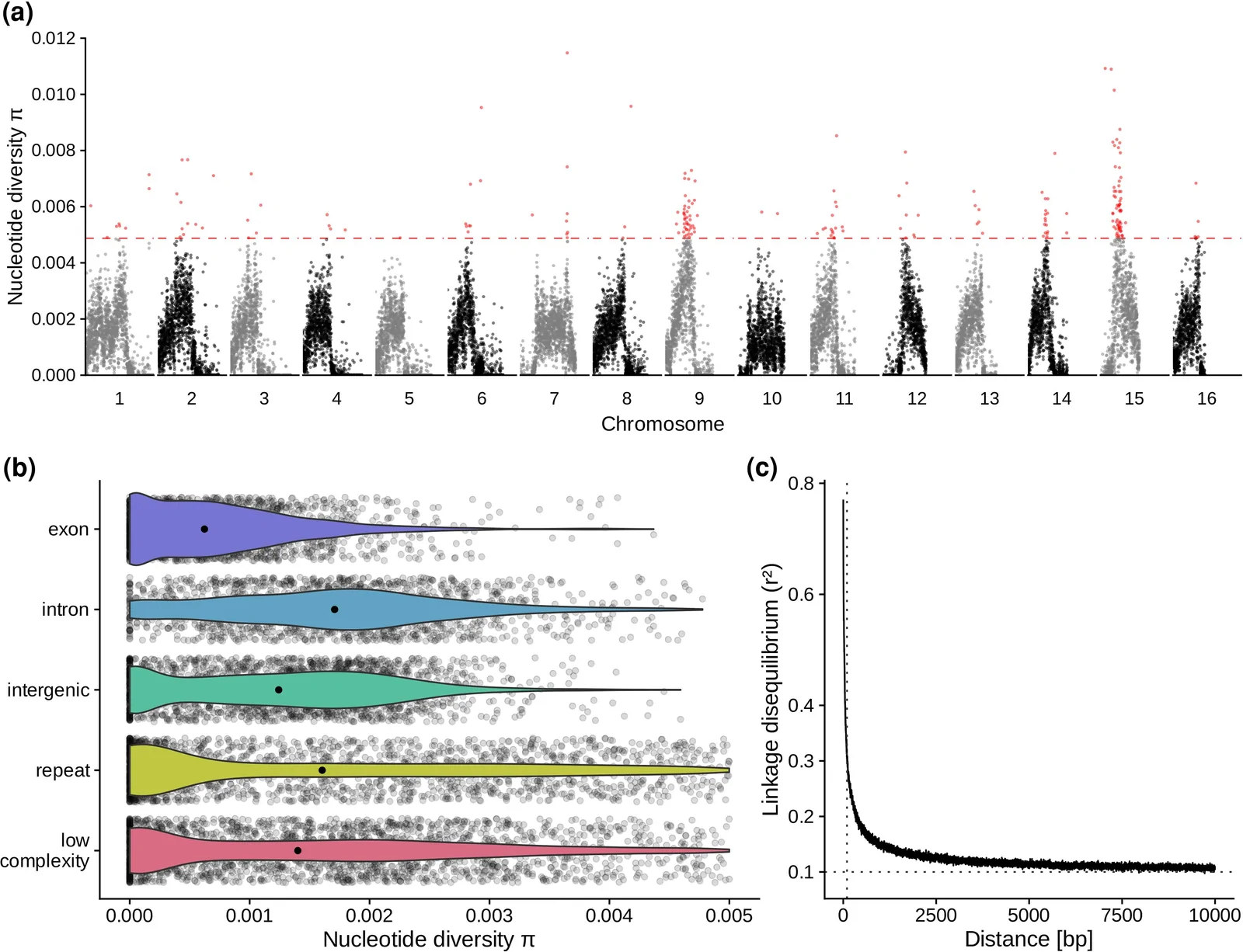
Low nucleotide diversity and low linkage disequilibrium decay inferred from whole-genome-resequencing. a) A Manhattan plot showing the distribution of nucleotide diversity estimates, as measured in π, along each of the 16 chromosomes of the *O. cornuta* genome assembly. To achieve a higher resolution, each dot represents a 10 kb window instead of the 100 kb window that we used to estimate the values reported in the text. Colored dots above the horizontal dashed line represent the top 5% most diverse windows across the whole-genome. b) Violin plots showing the distributions of nucleotide diversity estimates across primary genomic feature types with each feature type on a separate row and indicated by an individual color. The single black dots in the middle of each violin plot mark the median while transparent dots in the background show the raw data points (10 kb windows). c) A line graph showing the logarithmic decay of linkage disequilibrium (*y* axis) with increasing distance between loci (*x* axis). Linkage disequilibrium was computed in terms of *r*^2^. A dotted horizontal line marks the value of 0.1, which is the value of convergence for this curve, whereas a vertical dotted line marks a distance of 100 bp that can be used to compare LD decay across species. Here, at 100 bp, LD has decayed to a value of around 0.3.

Using our set of called variants, we calculated genome-wide estimates of nucleotide diversity in nonoverlapping 10 kb-sized windows finding a median diversity of π = 0.0012 (Fig. 4a) as well as a median Watterson's theta of θ_w_ = 0.001 across all 16 chromosomal scaffolds. This is considerably lower than whole-genome sequencing-based published estimates of nucleotide diversity in eusocial species of the genera *Apis* and *Bombus* (Colgan et al. 2022; Heraghty et al. 2023; Leroy et al. 2024; Wang et al. 2024), which range from π = 0.0014 to 0.0025 but is also lower than the estimate inferred for another solitary species of the same family (Megachilidae: *Megachile rotundata*) at θ_w_ = 0.003 (Jones et al. 2019). However, we are aware of the possibility that our relatively low sample size might capture less genetic variation and thus underestimate genetic diversity compared to other studies of higher sample size. We also identified heterogeneity in the chromosomal distributions of nucleotide diversity (Fig. 4b). Two chromosomal scaffolds (9 and 15) showed significant overrepresentation among the top 5% of windows in terms of elevated nucleotide diversity compared to the genome-wide background (Fisher's exact test, Benjamini–Hochberg adjusted *P* < 0.01). We also assessed nucleotide diversity differences across specific genomic regions and found that median nucleotide diversity was significantly elevated in both intronic (π *=* 0.0017) and repeat regions (π *=* 0.0016) compared to the rest of the genome (π *=* 0.0012, Wilcoxon rank-sum test, *P* < 1 × 10^−16^), but was not significantly elevated or decreased in intergenic (π *=* 0.0012) or low complexity (π *=* 0.0014) regions. In contrast, exonic regions (π *=* 0.0006), were significantly decreased in diversity compared to the rest of the genome (Wilcoxon rank-sum test, *P* < 1 × 10^−16^), which, especially compared to the three times higher diversity estimates found in introns, may be interpreted as potential evidence of different selective pressures acting on coding and noncoding regions of the genome (Fig. 4a). Linkage disequilibrium (LD) analysis revealed comparatively slow LD decay across the genome (Fig. 4c). The correlation coefficient (*r*^2^) stayed at values above 0.3 for distances below 100 bp and converged to around 0.1 by 10 kb of distance separating linked loci, whereas in another study on another species from the same family (Megachilidae: *M. rotundata*), LD decay reached values as low as around 0.05 by 10 kb of distance (Jones et al. 2019). In the same study, the recombination rate for *M. rotundata* was reported to be 1.02 cM/Mb. In contrast, the advanced eusocial western honeybee (Apidae: *A. mellifera*) reached LD levels as low as 0.05 already at distances below 100 bp due to its extremely high recombination rate of 23.94 cM/Mb (Jones et al. 2019). Together, our preliminary assessment provides evidence of reduced nucleotide diversity, albeit with peaks of elevated genetic diversity (Fig. 4), and relatively low rates of recombination both at shorter and longer physical distances in *O. cornuta* compared to social honeybees (23.94 cM/Mb), bumblebees (4.76 to 8.7 cM/Mb), stingless bees (9.3 to 12.5 cM/Mb), and solitary leafcutter bees (1.02 cM/Mb) (Stolle et al. 2011; Liu et al. 2017; Jones et al. 2019; Waiker et al. 2021).

**Fig. 5.**
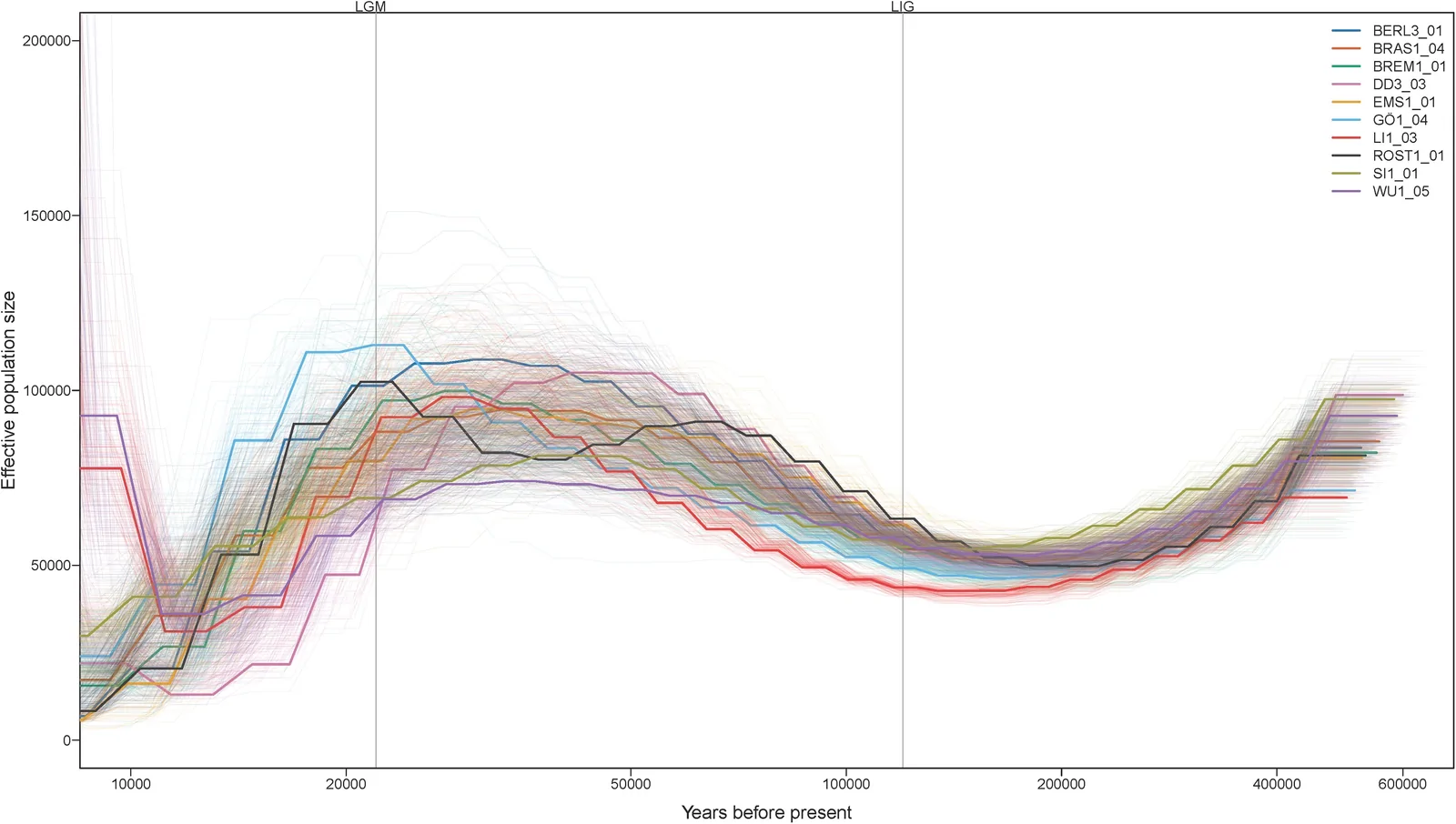
Effective population size is estimated to have declined since the Last Glacial Maximum. Line plot showing inferred demographic history of ten female *O. cornuta* sampled across Germany. The *y* axis represents effective population size, while the *x* axis indicates time in years before present. Each individual is represented by a distinct color, with faint lines illustrating the variation in effective population size estimates based on 100 bootstrap iterations. Vertical gray lines correspond to the Last Glacial Maximum (LGM) and Last Interglacial (LIG) period, respectively.

From our WGS data, we also inferred historic effective population sizes for *O. cornuta*, which suggest a decline toward and after the last glacial maximum (Fig. 5). For more recent historic times, the effective population sizes were consistent for samples between 10,000 and 30,000 with exception of two samples. Given that *O. cornuta* is currently undergoing an expansion toward the north of Germany (Westrich 2019), it is possible that the herein observed low genetic diversity in *O. cornuta* is attributable to reduced effective population size commonly observed at the forefront of a populations range expansion (Hartl and Clark 2007). Similarly, the reduced effective population size due to a historic population bottleneck, is an additional possible explanation for the observed reduced genetic diversity. However, these hypotheses need to be tested against data with higher sample size and from more established populations with less variable effective population size, such as those from glacial refugia. Furthermore, effective population size estimates are sensitive to demographic parameters, such as recombination rate and mutation rate, which are typically derived from closely related species. Given this reliance on reference data from other species, species-specific parameters for *O. cornuta* would substantially improve the reliability of population size estimates.

## Conclusion

Our study presents a highly contiguous, chromosome-level genome assembly, complete with a high-quality genome annotation for the ecologically and commercially important solitary bee *O. cornuta*. Given the lack of genomic resources that exist for *O. cornuta*, as well as other mason bees, this genome assembly provides a valuable resource for future genetic and genomic analyses to inform on how evolutionary processes are shaping their capacity to adapt to environmental pressures. We provide a demonstration of this potential by detailing preliminary measures of genetic variation, recombination rates, genome-wide methylation, as well as demographic changes within a European population. For example, using this dataset we found evidence of reduced genetic variation and reduced DNA methylation levels, which, together with this species’ current expansion of range and population levels, raises interesting questions about its potential for adaptation to future environmental challenges. Our project also demonstrates the feasibility to generate chromosome-level assemblies of high completeness *and* genome-wide DNA methylation profiles using as little as half of an Oxford Nanopore PromethION flow cell, which has an estimated cost of ∼€500, providing growing support to how improvements in sequencing costs and throughput, base calling accuracy, and assembly algorithms have increased the utility of Nanopore-sequenced long reads alone, to generate high-quality genome assemblies. Additionally, our study shows that a combined approach to genome annotation employing both transcriptomic- and orthology-based methods can improve the completeness of genome annotations. By providing both a resource for future analyses on an important solitary bee species but also novel, cost-effective approaches to genome assembly and annotation, we aim to contribute to an urgently needed, growing research interest in the genomics of understudied pollinators.

## Materials and Methods

### Sample Collection

For the purpose of genome assembly, in March 2023, one male adult was collected and immediately snap-frozen after emergence from pre-placed nest boxes from Rottleberode (Südharz, Saxony-Anhalt, Germany, 51°31′N, 10°57′E). For the purposes of gene model prediction, we sampled from adult and developmental stages and later complemented these with additional publicly available data to increase gene discovery rates, as certain genes may only be expressed during certain stages or tissues. We collected the following individuals: (i) one female adult was sampled from the same nest box as the male used for genome assembly and immediately snap-frozen after emergence; and (ii) male and female pupae (*n* = 30) were sampled in 2023 and 2024 from additional pre-placed nest boxes, and immediately snap-frozen. All samples were stored at −80 °C until DNA and RNA extractions were performed, respectively. Lastly, for the purposes of examining intraspecific genetic variation present within a representative *O. cornuta* population, we sampled female adults from 10 independent sites (more than 50 km apart) across Germany (for exact locations, see Table S1) for whole-genome resequencing (*n* = 10 individuals total) and downstream population genetic-based analyses. For sampled individuals, field-based species identification was initially performed based on morphological features (Westrich 2019), before bees were immediately conserved in absolute ethanol in the field with subsequent storage at −80 °C. Species identification for all samples was later assessed and validated using computational techniques (Supplementary Information: Computational validation of species identification).

### DNA Extraction and Long-Read Sequencing

High-molecular-weight DNA was extracted from the thorax of the single adult male using the New England Biosystems Monarch HMW DNA extraction kit, yielding 7.5 µg of DNA (260/280 nm ratio of 1.7, 230/280 nm ratio of 1.0, at a concentration of 150 ng/µl according to a Nanodrop ND-1000). A Nanopore sequencing library was generated using a ligation-based kit (SQK-LSK114) from Oxford Nanopore Technologies (ONT). Sequencing was performed on approximately half of the capacity of a PromethION flow cell (v10.4.1, FLO-PRO114M) in two runs of 20 and 48 h, respectively, with each run separated by a flow cell wash. The initial 39.57 Gb of raw data (4.86 million reads at N50 of 17 kb) were subjected to basecalling a second time with dorado (v0.7.0+71cc744, model sup5.0.0) on a Geforce GTX3090 GPU. Reads were then filtered by quality (≥Q90) and length (≥8 kb) (Filtlong v0.2.1, https://github.com/rrwick/Filtlong, seqtk v1.4-r122, https://github.com/lh3/seqtk), and, additionally, error-corrected with HERRO model_v0.1 for R10.4.1 data (Stanojevic et al. 2024) after which reads less than 5 kb were removed.

### RNA Extractions for Transcriptome-Facilitated Genome Annotation

For the prediction of gene models during genome annotation, we generated a transcriptomic dataset for the head tissues of a single *O. cornuta* adult female. To achieve this, from a snap-frozen sample, we removed the head from the thorax, and transferred it to a 1.5-ml centrifuge tube containing 200 μl of TRIzol. The sample was manually homogenized using a micropestle until the head was visually disrupted. Total RNA was subsequently extracted using a TRIzol-chloroform-based extraction protocol (Colgan et al. 2011), and then purified using a Monarch RNA purification kit (NEB) before being DNase-treated (TURBO DNase, Qiagen) to remove putative DNA contaminants. RNA was quantified and assessed using the FastGene NanoSpec Photometer (FG-NP01), before being stored at −80 °C. DNase-treated RNA was shipped on dry ice to a commercial supplier (Novogene, Germany) where quality was further assessed using an Agilent Bioanalyzer. An mRNA-enriched library was prepared and paired-end sequencing (2*150 bp) was performed to produce a minimum of 50 million paired-end reads. For the pupal samples, total RNA was extracted from four different leg tissues (hind tibia, mid tibia, hind basitarsus, mid basitarsus) from 30 samples (15 female, 15 male) using mechanical homogenization (Tissue Lyser LT, Qiagen) and a Zymo DirectZol kit, which included DNAse digestion. For the female adult, a mRNA-enriched library was prepared, while for the pupae samples mRNA was extracted from total RNA (poly-T oligo-attached magnetic beads), followed by fragmentation and cDNA synthesis with random hexamer primers. Sequencing (paired end 150 bp) was performed by Novogene (Germany) generating a mean read count of 55,206,130 across samples (min: 12,042,090, max: 162,207,301).

### DNA Extractions for Population Genetic Analyses

For the population genetic-based analyses, we removed ethanol-preserved samples (*n* = 10) from the −80 °C freezer. As abdomens can harbor microbial contaminants, we removed them using microscissors and the remaining tissue was subsequently washed with three rounds of autoclaved phosphate-buffered saline (PBS) to remove residual ethanol. Tissue homogenization was performed using micropestles on tissue samples in 1.5-ml Eppendorf tubes filled with 200 µl of PBS. Following this, DNA was extracted using the Monarch Genomic DNA Purification kit, according to the manufacturer's instructions. After extraction, the quality and quantity of isolated genomic DNA were checked using NanoDrop and Qubit photometric measurements, as well as by using low concentration agarose gel electrophoresis at low voltage. Only samples with a minimum total DNA amount of 1.2 µg, 260 nm:280 nm absorbance spectrum ratios of at least 1.8, and visible sharp bands in electrophoresis gels were considered for sequencing. Extracted DNA was sent to a commercial sequencing company (Novogene, UK) where further quality assessments using an Agilent Bioanalyzer were performed. After quality checks, for each sample, an individual PCR-free library was produced using a NEB library kit, individually barcoded, and sequenced on an Illumina NovaSeq 6000 (2*150 bp) to a minimum sequencing output of 3 Gb per sample, resulting in a mean read count of 32,695,578 across samples (min: 26,511,816, max: 38,517,412, Table S1), as well as a mean sequencing depth of 19×, based on the total length of chromosomal scaffolds.

### Genome Assembly

Using a combination of filtered long FASTQ reads and HERRO-corrected FASTA reads, we generated and assessed different de novo assemblies using flye v2.9.3 (Kolmogorov et al. 2019), specifically, generating assemblies with variation in minimum overlap (10,000, 8,000, and 6,000 bp) with presets for pacbio-hifi (corrected reads), nano-corr (corrected reads), and nano-hq (un-corrected reads). As a complementary approach, we generated a second set of assemblies using hifiasm v0.24.0 (Cheng et al. 2024) using the “–ont” flag with uncorrected reads and with five different parameter sets (see code on GitHub repository for this study). From each resulting assembly, we removed contigs shorter than 5 kb using seqtk as long reads used for the assemblies were either 8 kb or longer meaning that contigs shorter than this length represent potential sequencing artifacts or are biologically less informative. Each assembly was assessed for summary statistics (assembly_stats, https://github.com/sanger-pathogens/assembly-stats), gene completeness (Compleasm v0.2.7 with database hymenoptera_odb12), and the presence of the canonical hymenopteran telomere repeat “AACCT” using tidk (Brown et al. 2025).

The best assembly was assessed and chosen according to the lowest number of missing or duplicated BUSCO (Manni et al. 2021) genes, the highest number of identified telomeric regions, and the longest contigs. Based on these criteria, “hifiasm3” was selected as the best assembly, which was generated using hifiasm with the following parameters: “–telo-m AACCT –primary -l 2 -s 0.65 -D 10.0 -N 300 –dual-scaf –ont.” This assembly was polished using medaka v1.12.1 (https://github.com/nanoporetech/medaka), followed by the purging of haplotigs and overlaps using purge_dups v.1.2.5 (Guan et al. 2020), as well as the sorting by length in a descending order (ie longest to shortest) and renaming of contigs using seqkit v2.5.1 (Shen et al. 2016). The quality of the assembly was then again assessed using assembly_stats, Compleasm, and tidk, which collectively provide information on basic summary statistics of genome size and structure, assembly completeness, and potential contaminants. As an additional step, assembly completeness relative to the long reads used for assembly was assessed with merqury (version 2022-09-07 from the Github master at https://github.com/marbl/merqury; Rhie et al. 2020).

The putative mitochondrion was assembled in a separate process. First, we used BLASTN (Camacho et al. 2009) with a set of bee mitogenomes to identify six contigs likely of mitochondrial origin. One of these contigs, representing a putative single mitochondrial genome copy, was selected as a reference so as to allow extraction of Nanopore long reads that matched it after mapping with minimap2 v2.28-r1209 (Li 2018). These extracted reads were subsequently assembled using flye v2.9.3-b1797 (Kolmogorov et al. 2019) into a single contig of 35,525 bp. We used Blastn of contig ends and Minimap alignment to itself to confirm that the ends are not overlapping. This assigned mitogenome was then annotated with mitos v2 (Donath et al. 2019). To confirm that no eukaryotic genes are present on the mitochondrial contig, we used galba (Brůna et al. 2023) and helixer (https://www.plabipd.de/helixer_main.html).

### Assessment of Possible Contamination

We screened the assembled nuclear genome using BlobToolKit (Challis et al. 2020) to identify scaffolds of unusual GC content-to-read coverage ratios, as well as scaffolds assigned to nontarget organisms, which likely represent contaminants. In addition, the assembly was further assessed for contamination using the NCBI Foreign Contamination Screen (fcs v0.4.0; O’Leary et al. 2024) pipeline with NCBI's *O. cornuta* TaxID 185587 specifically selected.

### Repeat Annotation

Putative repetitive elements in the genome assembly were identified and annotated using EarlGrey v5.1.1 (Baril et al. 2024), which incorporates RepeatMasker v4.15 (https://github.com/Dfam-consortium/RepeatMasker) with the DFAM v3.7 and RepBase (2018) databases, and was run with clustering and softmasked output. This soft-masked genome assembly was used as the input for gene model predictions (“Gene annotation”). Satellites were identified with Satellite Repeat Finder (https://github.com/lh3/srf), while microsatellites and regions of low complexity were identified using a combination of the following complementary tools: etrf (-m 200 -l 13; https://github.com/lh3/etrf), trf-mod v4.10.0, SA-SSR v0.1 alpha (−min-repeats 5 –min-nucs 10 –max-ssr-len 12 max-seq-len 25000000), ULTRA v1.0.0 beta 12 (−min_unit 4 –min_length 10 –period 300, filtered for minimum length of 100 bp), tantan v49 (-f4, filtered for minimum repeat number = 5), sdust (-w 64 -t 20, filtered for minimum length = 10), and hrun (filtered for minimum length = 10, seqtk v1.3-r115-dirty). Low mappability regions were identified using genmap v1.3.0 (parameters: -K 35 -E 2), with subsequent filtering based on mappability below 0.05 and a minimum length of 100 bp. From the EarlGrey annotations, we removed entries classified as “Unknown” and “Satellite” to create a set of transposable element (TE) annotations. Putative microsatellite and low complexity regions were merged and overlaps with TE annotations removed using bedtools to create a set of repeat annotations.

### Gene Annotation

To increase our ability at predicting gene models for tissue-specific or lowly-expressed genes, we complemented our samples of adult head (*n* = 1) and pupal leg (*n* = 30) tissue with publicly available samples of antennal tissue (*n* = 8) generated by the Institute of Applied Genomics (NCBI BioProject: PRJNA185987), as well as a publicly available whole-bodied sample generated through the 1KITE project (*n* = 1, NCBI BioProject: PRJNA252331). Prior to gene model prediction, we performed data quality evaluations for all samples. For each sample, we first examined quality metrics generated using FastQC (Andrews 2010), which provides information on base quality, as well as presence and extent of adapter contamination. As a complementary approach, we investigated the proportion of raw reads mapping to the generated genome assembly using HISAT v2.2.1 (Kim et al. 2019), finding high mapping rates (mean: 96.06%). We then combined and visualized the outputs for both sets of quality assessments using MultiQC v.1.29 (Ewels et al. 2016). Based on the results of the quality assessment, we removed adapter sequences, which can affect mapping rates and quality, and filtered reads by quality (PHRED score ≥ 15) and length (minimum length ≥ 50 bp) using fastp v.0.20.1 (Chen et al. 2018).

The filtered reads were then used in conjunction with a protein database comprising arthropod-derived proteins from OrthoDB v12 (Kuznetsov et al. 2023), published hymenopteran proteins (Kapheim et al. 2015), and all proteins from the curated Swiss-Prot database (UniProt Consortium 2023) to predict gene models within the soft-masked genome assembly using BRAKER3 v3.0.8 (Gabriel et al. 2024). Initial predictions from this approach were further processed to identify and correct putative incorrect gene models, such as by splitting up gene models with no overlap among their transcript “children” into separate gene models, and by removing transcripts with internal stop codons or missing start or stop codons. As a complementary approach, gene models were independently predicted via a second approach based upon orthology to the *O. bicornis* reference genome (iOsmBic2.1, GCF_907164935.1) using TOGA v1.1.7 (Kirilenko et al. 2023). The resulting predictions, based upon orthologous matches, were filtered to include only predictions classified as “intact,” “partially intact,” or “uncertain loss.” Genes with no overlap among any of their transcripts were split into separate genes. During gene model prediction, when transcripts from *O. bicornis* were aligned to the *O. cornuta* genome, some transcripts mapped to different chromosomal scaffolds or contigs than the rest of the transcripts from the same source gene. These inconsistently mapped transcripts were removed to make sure that each newly predicted gene model in *O. cornuta* was single locus. As the orthology approach to gene prediction sometimes generated very short introns of 1 to 3 bp in otherwise correctly predicted transcripts, we joined adjacent exons if they were separated by less than 4 bp. Incomplete gene predictions with missing start or stop codons, or internal stop codons, were removed to retain only the highest quality predictions. We then computed overlap statistics between the two sets of annotations and complemented the BRAKER3 gene models with any additional transcript isoforms that were unique to the filtered TOGA predictions.

We inferred functional annotation from our translated, predicted proteins via homology and structural similarity to the publicly available annotation databases InterPro, Egg-Nog, BUSCO, dbCAN, SignalP, and UniProt (Simão et al. 2015; Almagro Armenteros et al. 2019; Huerta-Cepas et al. 2019; Blum et al. 2021; Zheng et al. 2023; UniProt Consortium 2025) using Funannotate v1.8.17 (Palmer and Stajich 2020). Lastly, we quality checked our final genome annotation using Compleasm in “protein” mode (Manni et al. 2021; Nevers et al. 2025) and OMark v0.3.1 (Nevers et al. 2025). To complement our annotation with tRNA and rRNA gene models, we ran tRNAscan-SE (v2.0.12; Chan et al. 2021) and Barrnap (v0.8, eukaryotic mode: -k euk; Seemann n.d.), respectively. Finally, putative mitochondrial fragments integrated into the genome (ie NUMTs) were assessed by querying the *O. cornuta* nuclear genome assembly using BLASTN with the putative mitochondrial genome sequence previously identified in our assembly.

### Methylation Analysis

As methylation can play an important role in gene regulation, we examined our Nanopore data for base modifications to identify presence and extent of methylation patterns in the *O. cornuta* genome. We used dorado v0.9.0 (https://github.com/nanoporetech/dorado) to basecall the raw Nanopore data (pod5) with the base modifications 5mCG, 5hmCG, 6mA, 5mC, and 5hmC, using the super accurate model (dna_r10.4.1_e8.2_400bps_sup@v4.3.0) on a Geforce RTX3090 (with parameter –modified-bases-threshold 0.30). We extracted base modification signals using modkit pileup (v0.4.1, https://github.com/nanoporetech/modkit) from the resulting sorted BAM file. To assess how methylation patterns differed among genomic regions and features (eg exonic, intronic, intergenic), we first counted, for each of these feature types, the total number of sites that could potentially be methylated using bedtools intersect. Similarly, we then counted for each feature type, the number of sites that were either weakly methylated (sites covered by at least one read containing a methylated base) or highly methylated (sites with at least 20% of reads containing a methylated base). To account for large differences in the mean length of features, we summarized all counts in 100 kb windows (bedtools map -o sum) across the genome, before dividing counts of weakly and highly methylated sites by the total number of sites that could potentially be methylated to generate percentages of methylation across all feature types. Throughout this approach, we processed 5mCG and 5hmCG methylation signals separately.

### Analysis of Synteny With *O. Bicornis*

To further assess contiguity and completeness of our genome assembly, we examined collinearity, particularly synteny of chromosomal segments, between our *O. cornuta* genome assembly and a reference genome assembly of the closely related red mason bee, *O. bicornis*. To this end, we extracted the longest predicted protein isoforms from both assemblies and ran a protein-to-protein BLAST (BLASTP) search (Camacho et al. 2009) to identify putative homologs. The result of this BLAST search was then provided as input into MCScanX v1.0.0 (Wang et al. 2012), which identified collinear blocks between the two assemblies that were subsequently visualized using the web application SynVisio (Bandi and Gutwin 2020).

### Calling, Filtering, and Annotation of Variants

Using the same quality control (QC) pipeline as that for the RNA-seq data, we assessed the raw sequencing data for the short-read whole-genome resequencing (WGS) samples for read-based quality metrics, as well as read mapping rates against our annotated genome assembly using bwa-mem v2.2.1 (Li 2013). Summarization of mapping statistics was performed using samtools stats (Danecek et al. 2021) and the generation of overview visualizations using MultiQC (Ewels et al. 2016). Raw reads were quality filtered and adapters trimmed using fastp (Chen et al. 2018) with quality (PHRED score ≥ 15) and length (minimum length ≥ 50 bp) filters. For variant calling, filtered reads were first mapped against the genome assembly using bowtie2 v2.5.4 (Langmead and Salzberg 2012), followed by variant calling using the BCFtools v1.21 mpileup-call pipeline (Danecek et al. 2021). Variants were filtered using VCFtools v0.1.17 (Danecek et al. 2011) with the following parameters: removal of indels; minimum and maximum mean read depth filters of 5 and 500, respectively; and a minimum quality score of 40. Additionally, a maximum of 20% of samples (ie two individuals) per site was allowed to have missing data. After filtering, the mean and median quality (QUAL) scores were 576.2 and 389, respectively. Filtered variants were used as input to a principal component analysis to investigate the genetic relationship between samples (Fig. S1). Lastly, variants were annotated with SnpEff v5.2f (Cingolani et al. 2012) using protein-coding sequence predictions from our annotation pipeline.

### Inference of Nucleotide Diversity and Linkage Disequilibrium

Filtered variants were indexed using tabix (Li 2011) before running pixy v1.2.11 (Korunes and Samuk 2021) to infer nucleotide diversity π and Watterson's θ across all chromosomes in both 100,000 and 10,000 bp sliding windows. In addition to a sliding window approach, π was inferred collectively for each investigated genomic feature (exon, intron, intergenic regions, repeats, and low complexity regions) to understand how different functional regions of the genome differ in nucleotide diversity. We inferred linkage disequilibrium decay curves from filtered variant calls using PopLDDecay v3.43 (Zhang et al. 2019). Inbreeding coefficients for population genomics samples were calculated using VCFtools –het.

### Demography Inference

To infer past changes of effective population size, we applied PSMC (Li and Durbin 2011) to sequencing data from the 10 sequenced female adults (Table S1). Variants were called and consensus sequences generated employing a pipeline that consisted of samtools v1.08 mpileup, bcftools v1.10.2 call, and vcfutils.pl (the “vcf2fq” pipeline). To improve variant detection in low coverage samples we copied and merged forward and reverse reads. For PSMC, we used parameters informed by related species. Based on estimations from the buff-tailed bumblebee (Apidae: *B. terrestris*), a mutation rate (μ) of 3.6 × 10^−9^ (Liu et al. 2017) per base pair per generation was chosen, assuming one generation per year as the species is univoltine (Tasei and Picart 1973; Splitt et al. 2022). Additionally, the recombination rate estimates for a closely related solitary bee (Megachilidae: *M. rotundata*; Jones et al. 2019), which is 1.02 cM/Mb and equivalent to a per base recombination rate of 1.02 × 10^−8^, was used, producing a recombination to mutation rate ratio of 2.5 (-r). We applied a maximum TMRCA of −7 (-t) and the atomic time interval pattern was set to “2 + 2 + 25*2 + 4 + 6” (-p), to increase comparability (Hilgers et al. 2025). To assess variance in effective population size estimates, we performed 100 bootstrap replicates per individual.

## Supplementary Material

evaf224_Supplementary_Data

## Data Availability

The genome assembly, as well as scripts required for reanalysis, are provided at https://github.com/LIB-insect-comparative-genomics/Osmia.cornuta.genome. The genome assembly and long-read fastq reads are publicly available on NCBI under the BioProject accession PRJNA1289724. DNA resequencing and RNA data are available under the BioProject accession PRJNA1289047. Additional RNA data from pupal leg tissue are publicly available as a hintfile (.gff) in the GitHub repository.
