# Correction: A Chromosome-Level Genome Assembly and Resequencing Data Reveal Low DNA Methylation and Reduced Diversity in the Solitary Bee Pollinator *Osmia cornuta*

**DOI:** 10.1093/gbe/evag210

**Published:** 2026-09-02

This is a correction to: Jannik S Möllmann, Xuejing Hu, Eva A Baumgarten, Anne Hartleib, Katja Nowick, Thomas J Colgan, Eckart Stolle, A Chromosome-Level Genome Assembly and Resequencing Data Reveal Low DNA Methylation and Reduced Diversity in the Solitary Bee Pollinator *Osmia cornuta*, *Genome Biology and Evolution*, Volume 18, Issue 1, January 2026, evaf224, https://doi.org/10.1093/gbe/evaf224

In the originally published version of the paper an errored link was published under the Data availability section.

This is now corrected to read:

"The genome assembly, as well as scripts required for reanalysis, are provided at https://github.com/LIB-insect-comparative-genomics/Osmia.cornuta.genome. The genome assembly and long-read fastq reads are publicly available on NCBI under the BioProject accession PRJNA1289724. DNA resequencing and RNA data are available under the BioProject accession PRJNA1289047. Additional RNA data from pupal leg tissue are publicly available as a hintfile (.gff) in the GitHub repository."

instead of:

"Data and scripts required for reanalysis are provided as supplementary files and will be made publicly available on GitHub and Zenodo upon acceptance. Currently, for the purpose of reviewing, all supplementary information can be retrieved from the following link: https://drive.google.com/drive/folders/1-eYz0K6re6_K9PQap43zA81LMbrUK6hl?usp=sharing. The genome assembly and long-read fastq reads will be made publicly available on NCBI under the BioProject accession PRJNA1289724 upon publication. DNA resequencing and RNA data will be made available under the BioProject accession PRJNA1289047. Additional RNA data from pupal leg tissue will be made publicly available as hintfile (.gff) via Github and Zenodo upon publication."

The emendation has been made to the paper.

