# Supplementary material for: A Chromosome-Level Genome Assembly and Resequencing Data Reveal Low DNA Methylation and Reduced Diversity in the Solitary Bee Pollinator Osmia cornuta
Source: Genome Biol Evol. 2026 Jan 8;18(1):evaf224. doi: 10.1093/gbe/evaf224 (PMC12780776; doi:10.1093/gbe/evaf224)
Supplement: evaf224_Supplementary_Data [file evaf224_supplementary_data.pdf]

Supplementary Information

Computational validation of species identification

Species identification for all samples was initially performed based on morphological characteristics but later confirmed computationally. To that end, we first identified the locus of the *O. cornuta* cytochrome c oxidase subunit 1 (COX1) sequence within our genome assembly via orthology with the COX1 sequence of the *Osmia bicornis* reference genome (iOsmBic2.1, GCF_907164935.1) using BLASTN (v2.14.1+, (Camacho et al. 2009)). The *O. cornuta* COX1 orthologue sequence was then extracted from this locus using samtools faidx (Danecek et al. 2021) and searched against COX1 sequences housed in the database of Barcode of Life Data Systems (BOLD, queried on the 3rd of October, 2024, (Ratnasingham and Hebert 2007)), validating that the male, which we used to generate the genome assembly was correctly identified as *O. cornuta*. For all other DNA- and RNA-seq samples, reads were mapped against the *O. cornuta* genome assembly using STAR (v2.7.11a, (Dobin et al. 2013)) and for each sample a consensus sequence of matches against the identified *O. cornuta* COX1 locus was constructed using samtools consensus (Danecek et al. 2021). The resulting COX1 sequences were then searched against BOLD using their web API to confirm species identifications.

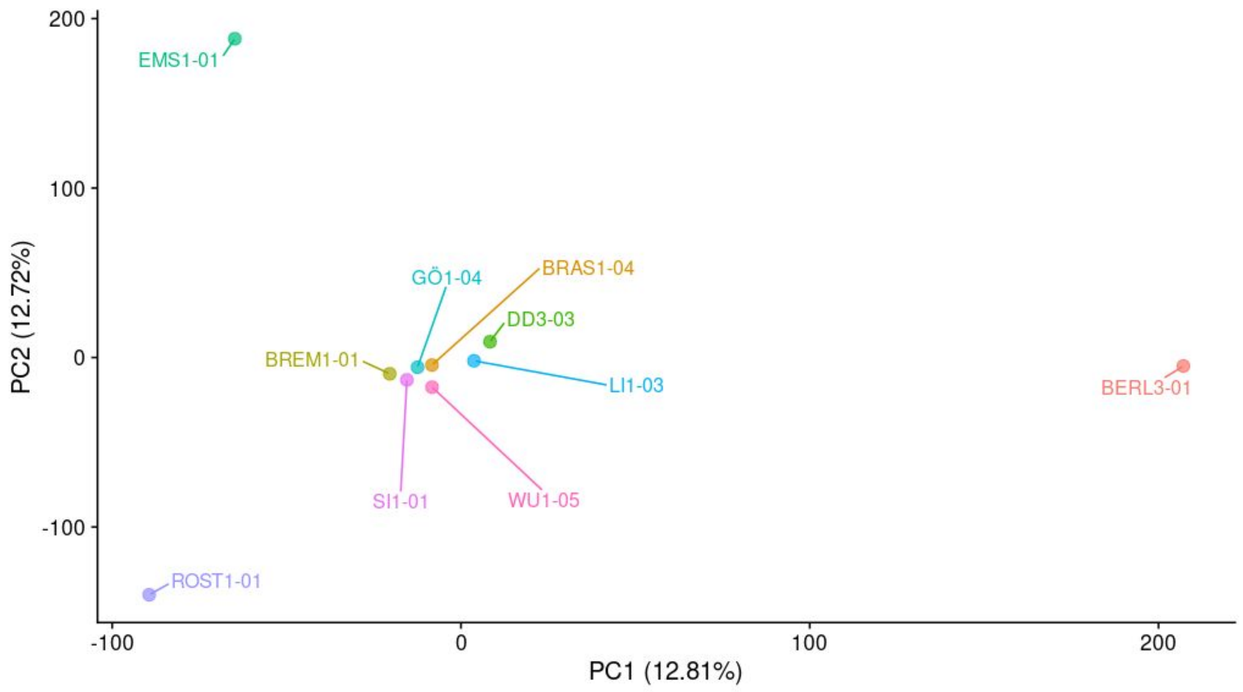

Supplementary Figure 1. Genetic relationship among sampled *Osmia cornuta*. Scatterplot displaying the first two principal components calculated from a principal component analysis based on single nucleotide polymorphisms called within a population of *Osmia cornuta* sampled across different locations in Germany. The x-axis shows the first principal component and the total variance explained by it, while the y-axis shows the second principal component and the variance it explains. Each coloured and labeled dot represents a single female *O. cornuta*.

Sample ID	Sampling Date	Latitude	Longitude	Altitude [m]	Inbreeding Coefficient F	Raw Read Count
BERL3-01	12/04/23	52.60	13.40	49	0.21189	38,517,412
BRAS1-04	29/03/23	52.31	10.52	69	0.10376	28,428,320
BREM1-01	10/04/23	53.07	8.80	8	0.19567	29,153,738
DD3-03	05/05/22	50.88	13.68	387	0.18829	36,106,378
EMS1-01	10/04/23	52.36	7.30	41	0.23890	36,025,098
GÖ1-04	23/04/22	48.71	9.63	338	0.11099	26,511,816
LI1-03	02/05/22	50.39	8.05	129	0.12830	31,449,700
ROST1-01	11/04/23	54.09	12.09	26	0.19660	34,304,438
SI1-01	23/04/22	49.22	8.79	197	0.04068	31,255,604
WU1-05	27/04/22	49.77	9.93	233	0.11870	35,203,272

Supplementary Table 1. Sample site information for *Osmia cornuta* used for population genomics. The table is showing for each *O. cornuta* that was considered for the population genomics analyses, the sample identifier, the date of sampling, sampling site coordinates, inbreeding estimates calculated from expected versus observed homozygosity, and the total count of raw reads generated for each sample.

Category	Osmia cornuta	Osmia lignaria	Osmia bicornis
Assembly level	Chromosome	Chromosome	Chromosome
Genome coverage	40.22	137	93
Assembly length [Mb]	647.7	498.5	223
Contig count	727	236	563
N50 [Mb]	3.94	14.2	9.1
GC content [%]	34.8	39	39.5
Gene count	12144	13795	12706
BUSCO completeness [%]	99.78	97.9	98.6

Supplementary Table 2. Comparison of assembly and annotation statistics with reference species. The table is showing assembly and annotation statistics across several categories for the herein presented genome for *Osmia cornuta*, in comparison to statistics from pre-published, high-quality RefSeq genomes for *Osmia bicornis* and *Osmia lignaria.*

References

Camacho, Christiam, George Coulouris, Vahram Avagyan, Ning Ma, Jason Papadopoulos, Kevin Bealer, and Thomas L. Madden. 2009. “BLAST+: Architecture and Applications.” *BMC Bioinformatics* 10 (1): 421. https://doi.org/10.1186/1471-2105-10-421.

Danecek, Petr, James K. Bonfield, Jennifer Liddle, John Marshall, Valeriu Ohan, Martin O. Pollard, Andrew Whitwham, et al. 2021. “Twelve Years of SAMtools and BCFtools.” *GigaScience* 10 (2). https://doi.org/10.1093/gigascience/giab008.

Dobin, Alexander, Carrie A. Davis, Felix Schlesinger, Jorg Drenkow, Chris Zaleski, Sonali Jha, Philippe Batut, Mark Chaisson, and Thomas R. Gingeras. 2013. “STAR: Ultrafast Universal RNA-Seq Aligner.” *Bioinformatics (Oxford, England)* 29 (1): 15–21. https://doi.org/10.1093/bioinformatics/bts635.

Ratnasingham, Sujeevan, and Paul D. N. Hebert. 2007. “Bold: The Barcode of Life Data System (http://www.barcodinglife.org).” *Molecular Ecology Notes* 7 (3): 355–64. https://doi.org/10.1111/j.1471-8286.2007.01678.x.
